# The 2006 NESCent Phyloinformatics Hackathon: A Field Report

**Published:** 2007-12-14

**Authors:** Hilmar Lapp, Sendu Bala, James P. Balhoff, Amy Bouck, Naohisa Goto, Mark Holder, Richard Holland, Alisha Holloway, Toshiaki Katayama, Paul O. Lewis, Aaron J. Mackey, Brian I. Osborne, William H. Piel, Sergei L. Kosakovsky Pond, Art F.Y. Poon, Wei-Gang Qiu, Jason E. Stajich, Arlin Stoltzfus, Tobias Thierer, Albert J. Vilella, Rutger A. Vos, Christian M. Zmasek, Derrick J. Zwickl, Todd J. Vision

**Affiliations:** 1 National Evolutionary Synthesis Center, 2024 W. Main St., Suite A200, Durham NC 27705, U.S.A; 2 Dunn Human Nutrition Unit, Medical Research Council, Hills Road, Cambridge CB2 0XY, United Kingdom; 3 Department of Biology, CB 3280, University of North Carolina, Chapel Hill, NC 27599; 4 Genome Information Research Center, Research Institute for Microbial Diseases, Osaka University, 3-1 Yamadaoka, Suita, Osaka 565-0871, Japan; 5 School of Computational Science, 150-F Dirac Science Library, Florida State University, Tallahas-see, Florida 32306-4120, U.S.A; 6 EMBL—European Bioinformatics Institute, Wellcome Trust Genome Campus, Hinxton, Cambridge, CB10 1SD, United Kingdom; 7 Section of Evolution and Ecology, Center for Population Biology, 3347 Storer Hall, University of California, Davis, CA 95616, U.S.A; 8 Human Genome Center, Institute of Medical Science, University of Tokyo, 4-6-1 Shirokanedai, Minato-ku, Tokyo 108-0071, Japan; 9 Department of Ecology and Evolutionary Biology, University of Connecticut, 75 North Eagleville Road, Unit 3043, Storrs, CT 06269-3043, U.S.A; 10 GlaxoSmithKline, 1250 S. Collegeville Road, Collegeville, PA 19426, U.S.A; 11 ZBio LLC, Nyack, NY 10960, U.S.A; 12 Peabody Museum of Natural History, Yale University, 170 Whitney Ave., New Haven CT 06511, U.S.A; 13 University of California, San Diego, Division of Comparative Pathology and Antiviral Research Center, 150 West Washington Street, San Diego, CA 92103; 14 Department of Biological Sciences, Hunter College, City University of New York, 695 Park Ave, New York, NY 10021, U.S.A; 15 Department of Plant and Microbial Biology, University of California, Berkeley, CA 94720, U.S.A; 16 Biochemical Science Division, National Institute of Standards and Technology, 100 Bureau Drive, Mail Stop 8310, Gaithersburg, MD, 20899-8310; 17 Biomatters Ltd, Level 6, 220 Queen St, Auckland, New Zealand; 18 Department of Zoology, University of British Columbia, #2370-6270 University Blvd., Vancouver, B.C. V6T 1Z4, Canada; 19 Burnham Institute for Medical Research, La Jolla, CA 92037, U.S.A; 20 Department of Biology, Duke University, P.O. Box 90338, Durham, NC 27708, U.S.A

**Keywords:** phylogenetics, phyloinformatics, open source software, analysis workflow

## Abstract

In December, 2006, a group of 26 software developers from some of the most widely used life science programming toolkits and phylogenetic software projects converged on Durham, North Carolina, for a Phyloinformatics Hackathon, an intense five-day collaborative software coding event sponsored by the National Evolutionary Synthesis Center (NESCent). The goal was to help researchers to integrate multiple phylogenetic software tools into automated workflows. Participants addressed deficiencies in interoperability between programs by implementing “glue code” and improving support for phylogenetic data exchange standards (particularly NEXUS) across the toolkits. The work was guided by use-cases compiled in advance by both developers and users, and the code was documented as it was developed. The resulting software is freely available for both users and developers through incorporation into the distributions of several widely-used open-source toolkits. We explain the motivation for the hackathon, how it was organized, and discuss some of the outcomes and lessons learned. We conclude that hackathons are an effective mode of solving problems in software interoperability and usability, and are underutilized in scientific software development.

## Introduction

There is a growing abundance of comparative biology data, motivating a wide diversity of studies that require the application of complex, multistep evolutionary analyses to many or large datasets (for example, Cliften et al. 2003; Stein et al. 2003; Demuth et al. 2006; Pollard et al. 2006; Bininda-Emonds et al. 2007; Sidlauskas, 2007). These studies depend on the ability to string together individual software components into workflows that can be easily reused and modified. The research community has produced a huge number of software components to choose from (e.g. Joe Felsenstein’s directory at http://evolution.genetics.washington.edu/phylip/software.html currently lists more than 300). This abundance of tools is both a blessing and a curse. For while they collectively enable a wealth of questions to be asked of comparative data, many of these programs are not compatible with one another due to a paucity of support for interoperability and data exchange standards. Even where common standards such as NEXUS (Maddison et al. 1997) are ostensibly supported, it is often in non-compliant ways (e.g. through the inclusion of undocumented extensions).

**Figure 1.**
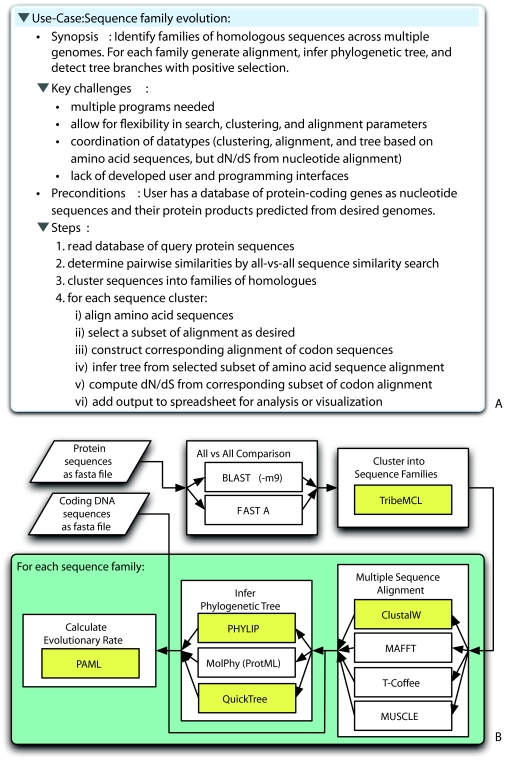
Sequence family evolution workflow. (**A**) Use-case description forming the basis of the workflow. (**B**) Schematic view of the workflow as implemented in the BioPerl package. The small inset boxes are labeled with the external programs integrated into the pipeline; multiple such boxes within a larger box indicate multiple alternatives supported by BioPerl for this step. For calculating evolutionary rates, BioPerl also supports mapping an amino acid alignment to a coding sequence alignment. Boxes shaded in yellow indicate programs with new or enhanced support in BioPerl due to work completed at the hackathon.

The basic workflow depicted in Figure 1 illustrates the kind of interoperability challenges that users routinely face. In this workflow, a large number of protein sequences are clustered into one or more sets of related sequences, the sequences within each cluster are then aligned, a phylogenetic tree is inferred for each alignment, and finally the rates of synonymous and non-synonymous substitutions are estimated for each branch of the tree in order to test for branches that may have experienced episodes of positive selection. While the homology search, clustering, and multiple alignment steps are performed using the protein sequences, estimation of substitution rates requires as input the alignment for the corresponding coding DNA sequences. However, the tools used for intermediate steps of the analysis may or may not have the capacity to pass the DNA and amino acid alignments along together, potentially leading to a break in the workflow. Although this problem can be solved with moderate effort, the difficulty is that every individual user has to solve it from scratch. As is inevitable with the many unrelated efforts in phylogenetic software development, a host of such interoperability gaps have arisen. These range from programs having different requirements for particular metadata fields (such as the length or use of quotes for taxon labels), to programs having structurally incompatible implementations of shared data exchange formats (such as the use of different blocks for the same datatype in NEXUS), to some programs that lack support for any common input or output standard at all. In addition to the redundant efforts expended by individual users in writing custom code to address these gaps in interoperability, barriers of this nature unnecessarily discourage other researchers from using phylogenetic analysis methods for comparative data analysis.

More than a decade ago, similar interoperability issues within the genome informatics community motivated the development of “glue code”, software written in a high-level language that can be used to invoke external programs with the appropriate parameters while shielding the user from the messy details of converting the output from one program into the input of the next (Stein, 1996). This strategy obviates the need to impose strict requirements for interface consistency and use of data exchange formats on analysis programs, and so can be applied retroactively to existing programs. The task of integrating a new program is reduced to the relatively simple problem of writing a wrapper for execution and handlers for the input and output data formats. High-level languages (e.g. Perl, Python, Ruby) facilitate this by robustly handling many of the mundane but error-prone tasks in parsing files, allocating memory and so on.

In 1995, BioPerl became the first general purpose toolkit that collected a large volume of glue code into a coherent, modular, and reusable package (Stajich et al. 2002). Since that time, a number of parallel and related efforts in other programming languages (e.g. Biojava, Biopython, and BioRuby) have been launched (Stajich and Lapp, 2006), collectively referred to as the Bio* toolkits (Mangalam, 2002). These projects, which are loosely affiliated under the umbrella of the Open Bioinformatics Foundation (http://www.open-bio.org/) are all freely available with licenses approved by the Open Source Initiative (OSI, see http://www.opensource.org), such as the Perl Artistic License for BioPerl (see the project websites for the specific license used by each project), and follow an open development model in which anyone may contribute new code. However, since the Bio* toolkits have each had unique histories and distinct emphases, they do not simply implement the same body of code in different languages, and they are at varying levels of maturity. As the Bio* projects to date have been driven largely by the needs of genome informatics, such as sequence analysis and genome annotation, there are substantial holes in support for phylogenetic analyses within these toolkits. Indeed, phylogenetic tools and data exchange standards are not widely known within the community of Bio* developers.

Recognizing the need for an improved informatics infrastucture for evolutionary analysis, the National Evolutionary Synthesis Center (NESCent) sponsored a “Phyloinformatics Hackathon” that took place December 11–15, 2006, at NESCent in Durham, North Carolina. A hackathon is an intense meeting in which the participants almost exclusively write software. The term was popularized by the OpenBSD community (see http://www.openbsd.org/hackathons.html) but is now widely used by open source developers. Hackathons are well suited to the development of community software resources because they foster direct face-to-face interactions and collaboration among participants. From a software development process viewpoint, this provides an environment highly conducive to many of the tenets of agile development (cf. Cockburn, 2002; Kane et al. 2006). For example, as participants literally program next to each other, pair programming and rapid feedback cycles for cross-checking design ideas arise organically, especially if the number and diversity of participants is chosen such that subgroups of four to five people form that work on a shared set of problems. The development loop from use case, requirement, prototype design, to testing and feedback can be closed on the spot, and the collective expertise reduces the chance for programming problems to remain “stuck”.

The goal of the NESCent Phyloinformatics Hackathon was to improve the level of interoperability and standards compliance in phyloinformatics by bringing together software developers from the Bio* toolkits and those from the phylogenetics community. While a hackathon format has been used successfully before by the Bio* toolkits (http://www.open-bio.org/wiki/Hackathon) and in other life science applications (for example SBML, http://www.sbml.org/wiki/HackathonNotes), to our knowledge, no large-scale hackathon had previously been held in the field of evolutionary informatics. Thus, it is appropriate to step back and consider the event as an experiment. How effective was the hackathon at strengthening the support for phylogenetic analysis tools and data types in the Bio* toolkits, and at making those tools amenable to seamless “plug-and-play” integration into automated workflows? What lessons were learned that could be used to guide future collaborative software development projects in evolutionary bioinformatics?

## Methods

An important question to address in planning the hackathon was how best to channel the efforts of the participants towards target problems that will have maximal impact. We chose to guide the selection of target problems by an examination of the gaps in desired analysis workflows as revealed by “use-cases” such as inferring divergence times from a phylogenetic analysis, or assigning duplications to a gene family tree, and so on. For our purposes, use-cases were generally informal descriptions of workflows, including the input data, the computational analysis steps required, and the desired end result. These were collected in advance on a public wiki from a variety of sources: an evolutionary informatics working group supported by NESCent (http://evoinfo.nescent.org/), hackathon participants, resident scientists at NESCent, and the evolutionary biology community at large (through a solicitation posted to evoldir, see http://evol.mcmaster.ca/brian/evoldir.html). The resulting set of 19 use-cases (see http://hackathon.nes-cent.org/UseCases) reflects a wide breadth of phylogenetic applications from organismal systematics to comparative biology to molecular evolution to genomics. An example of a use-case is given in Figure 1A; this example actually consolidates two of the original use-cases that were chosen as high-priority targets.

Hackathon participants were invited by the organizers based on nominations obtained through an open call to the evolutionary biology community (through a solicitation posted to evoldir), and the respective developer communities (through pertinent Bio* mailing lists). All participants had evident expertise in designing and programming reusable open source toolkits or phylogenetic analysis tools. The 26 attendees, from as far away as New Zealand and Japan, included software developers from the Bio* toolkits (BioPerl, Bio-java, Biopython, BioRuby, BioSQL; see Table 2) (Mangalam, 2002; Stajich et al. 2002), several phyloinformatics projects (Bio::NEXUS, Bio:: Phylo, NCL) (Lewis, 2003; Hladish et al. 2007), and some of the most widely used phylogenetic analysis programs (CIPRES, GARLI, HyPhy, PAUP) (Swofford, 2003; Pond et al. 2005; Zwickl, 2006) and databases (TreeBASE) (Piel et al. 2001). The group included programmers working in all the major programming languages in use in the life sciences (C, C++, Java, Perl, Python and Ruby). Selected participants from each of the Bio* toolkits came prepared to hold an initial “bootcamp” for new contributors. A bootcamp is essentially a short tutorial designed to help developers new to a tool-kit to get acquainted with its basic design and coding principles in order to enable them to contribute productively. One attendee was charged solely with documenting the target problems and the solutions that the participants implemented (see http://hackathon.nescent.org/Phylohackathon_1/Documentation). This was achieved in large part through “vignettes”, minimal but working code snippets that provide the user with ready-to-use demonstration programs that he or she can tinker with. Two software-literate evolutionary biologists served as floating “use-case stewards”; they were on call to answer questions from developers about the biological applications, to make sure the developers had appropriate test data, to test features, and to help with documentation.

The hackathon began with presentations by phylogenetics researchers on the perceived holes in phyloinformatics software infrastructure, and by software developers on existing efforts aimed at filling those holes. This led into a gap analysis of the use-cases that had been collected. The participants sought to identify what elements were missing that prevented successful implementation of a particular use-case from start to finish (e.g. data types not yet represented, or file formats not yet understood). The rationale was that by targeting these gaps, participants could ensure that their work would have an immediate and tangible impact by enabling a workflow of importance to researchers in the field.

Participants collectively consolidated and prioritized the use-cases, taking into account whether a gap represented an informatics infrastructure hole that could be immediately addressed by the participants in the room or whether it constituted an open research question that was beyond the scope of the hackathon, such as lack of a generally accepted algorithm solving the problem, or lack of an efficient implementation of it. This resulted in six problems chosen as development targets. Five represented concrete analysis workflows and included (i) the circumscription, alignment, phylogenetic analysis, and analysis of substitution rates for a gene or protein family starting with a database of sequences (also see Figure 1 for a graphical depiction of this workflow); (ii) reconciliation of a gene and species tree to determine patterns of orthology and paralogy; (iii) identification of highly conserved or fast-evolving sequence motifs through phylogenetic footprinting (Blanchette et al. 2002); (iv) inference of a phylogeny and support values using non-molecular characters; and (v) phylogenetic estimation of divergence times. In addition to these five problems, the recurring issue of compliance with the NEXUS data format standard was also judged to be of high priority and chosen as a sixth target.

Participants then broke into subgroups based on project affiliations and personal interests. Each subgroup chose its own targets to work on. In keeping with agile development principles (Cockburn, 2002), daily ‘stand-ups’ were held in which participants from each subgroup gave short progress reports in order to keep everyone informed of their activities and to collectively problem-solve when roadblocks were encountered.

## Results

The event produced a range of outcomes, both tangible and intangible, for both end-users and software developers. The most immediate result was the generation of many thousands of lines of software source code. Some participants committed over 5000 lines of code during the event, and much additional code has been written since. This code has been integrated into the software distributions of the respective open-source software projects. Thus, it is freely available under (several different) open source license agreements to the community of end-users and developers, and anyone can use or build upon it. The vignette-style documentation continues to be expanded to reflect ongoing development of the code base.

Instead of giving rise to new analysis tools, the outcomes improved the ability to seamlessly combine some of the most popular phylogenetic analysis programs into complex workflows. The breadth of the improvements is illustrated by the (noncomprehensive) list of targets and accomplishments given in Table 1. From the viewpoint of the end user, all of the participating Bio* toolkits substantially expanded their handling and coverage of data types and analyses commonly used in phylogenetics, as we illustrate with a few examples. The BioPerl group, which already had a phylogenetic data model to begin with, accomplished filling in all the gaps needed to obtain a complete workflow that starts with a database of unaligned sequences across genomes as input and yields as output gene or protein families and for each family the protein alignment, the inferred gene tree, and tree branches detected to be under positive selection (see Fig. 1). In addition, the group completed the workflows for phylogenetic footprinting (Blanchette et al. 2002) to detect functional genomic elements under selection, and for reconciling gene trees with species trees to infer gene duplication events. BioRuby, which had essentially no phylogenetic analysis coverage prior to the hackathon, now supports the NEXUS and Newick (http://evolution.genetics.washington.edu/phylip/newicktree.html) data formats, as well as several popular multiple sequence alignment programs. The BioSQL group created a relational model for trees or networks, implemented algorithms to precompute nested-set and transitive closure values for optimizing hierarchical queries, and defined a number of topological queries against the schema. Together, these constitute the foundation of an easily deployable standardized database of phylogenetic trees, and can be a building block for high-throughput applications that reconcile gene trees with species trees or determine concordance between different phylogenies. Among the CIPRES-affiliated projects, the Perl package Bio:: Phylo, which encapsulates phylogenetic tree manipulations and calculations, was made interoperable with the corresponding data model employed by the BioPerl toolkit. This comprises a first step towards making the functionality of BioPerl available to the CIPRES architecture (http://www.phylo.org/sub_sections/software.htm) (see also Moret, 2005), and vice versa. The participating HyPhy (Pond et al. 2005) developers collaborated with Biopython and BioPerl developers to add support for automatically running analyses in HyPhy using its batch execution language.

One cross-project subgroup made substantial progress on the issue of compliance with the NEXUS format. The group assembled a large collection of test files designed to expose common compliance issues. They also developed a proposal for defining levels of compliance, whereby a particular program can declare its input or output to be at a specified NEXUS compliance level, making its behavior (and possible failure) predictable when interacting with other programs that emit or consume NEXUS (see http://hackathon.nescent.org/Supporting_NEXUS). One immediate outcome of this effort was the adoption of an already existing reference implementation, NCL (Lewis, 2003), by the popular maximum-likelihood phylogenetic program GARLI (Zwickl, 2006) which had previously used its own much less robust implementation. This change will be available to the research community with the next release of GARLI (v0.96). Another immediate benefit was the improvement of NCL itself by correcting bugs revealed by the collected test files, and by adding a ‘normalizer’ application that converts a NEXUS-formatted input file to a NEXUS file that better follows recommended practices.

While the code is the most obvious outcome of the hackathon, it is far from the only one. Developers from different projects, communities, and backgrounds interacted intensely for five days working towards a common set of goals, and a number of productive new collaborations resulted from this stew. These cross-project relationships may, in the long-term, be more significant than the body of software code generated during the five day event. For instance, the hackathon spurred coordination between two major Java toolkits, Biojava and JEBL (http://sourceforge.net/projects/jebl/), which had previously been independent projects with seemingly irreconcilable design differences. Perhaps surprisingly, the willingness of the developers to collaborate on an interoperability layer obviated the need to unify the APIs. Similar to creating an interoperability bridge between the Bio::Phylo and BioPerl programming interfaces, this approach enables the user to take advantage of several software libraries if the problem benefits from doing so, without compromising the specific strengths of individual toolkits.

The hackathon also, for the first time, brought together the developers from CIPRES and the HyPhy software projects. These groups quickly realized that a common format for exchanging substitution models between programs was lacking and that multiple incompatible formats were already beginning to emerge. A common format understood by all programs would allow the researcher to distribute, rapidly evaluate, and easily reuse a model once it is defined. Taking advantage of the opportunity for extensive face-to-face discussions, the group created a first draft of such a standard, which is publicly available on the Hackathon website (see http://hackathon.nescent.org/CharacterModel_Object_Model).

To gauge how participants perceived the hackathon, its outcomes, and the possible effect of those on the interoperability landscape in phyloinformatics we conducted a brief survey of the participants shortly after the conclusion of the event (data not shown). Aside from feedback on logistics and infrastructure, the survey consisted of 19 questions about various aspects of planning the event, the utility of the use-case driven approach, and overall impact of the results. The responses were overwhelmingly positive (average of 4.0 on a scale from 1 to 5), and satisfaction with the outcomes and the perceived impact on the field received high ratings (average of 3.9 and 3.7, respectively).

## Discussion

From this experiment in scientific software development, we came away with a number of useful lessons to guide future efforts.

First, performing gap analysis and prioritizing use-cases is an effective means of focusing effort on high-impact development targets. Distinguishing open-ended research questions from those that can be addressed using existing tools requires the expertise of both developers and users, and so is best done prior to the hackathon itself. That said, acquiring use cases from the user community requires considerable effort and advance planning and not all use-cases are equally well suited for driving development targets. Furthermore, it may be necessary to further narrow down broad descriptions to recipe-like definitions when, as in our case, some participants lack scientific domain expertise. Additionally, while use-cases are excellent for jump-starting the work to be undertaken, the hackathon agenda needs to allow considerable flexibility. The intense interactions reveal unexpected issues and foster new ideas, and it would be counterproductive to constrain participants from pursuing these immediately. For example, the need to define an exchange standard for substitution models was not anticipated, but a subgroup could form spontaneously to address it.

Second, the appropriate mix of participants is important. While a key ingredient for success is the participation of experienced, highly-skilled software developers, it is also desirable to include those who are more novice. The abundance of experienced developers provides an excellent training environment, and an influx of new contributors is critical to the long-term success of any open-source software project. Along those same lines, we discovered that having some of the participants prepared to offer bootcamps, or short tutorials intended for developers new to a toolkit, enabled very productive cross-project interactions. For example, it enabled a HyPhy developer to add a HyPhy interface to the Biopython code base, and one of the creators of PhyloXML (http://www.phyloxml.org/) to contribute a NEXUS parser to the BioRuby project. Aside from the software developers, it is beneficial to have dedicated participants to entrust with the responsibility of writing useful software documentation, rather than relying on those who are writing the code and immersed in the technical details. Similarly, it can be valuable to have end-users on hand to provide expertise, assemble data, test code and review documentation. Our experience suggests that full utilization of this resource requires having scheduled interactions with each group rather than relying on those arising spontaneously.

Third, it is necessary to provide sufficient time and resources for the hackathon to be productive. The first day will invariably be spent setting the stage and discussing development targets. It takes another two to three days for developers to break down technical barriers for effective cross-project collaborations. Thus, we suggest that a hackathon should be planned to last at least four to five days. The technical infrastructure required for such an event is fairly straightforward: wireless network access, a mailing list, a wiki, and a shared filesystem are all desirable if not strictly necessary. It is generally not necessary to provide participants with personal computers. The infrastructure for dissemination of the code will depend on the circumstances. In our case, we found that most developers already used existing source code repositories (e.g. on Sourceforge) that are widely known by users and other developers, and so an event-specific source-code repository was not desirable. Once the event has concluded, regular follow-up effort is needed. The work that gets accomplished during the hackathon itself is seldom a finished product, and the clean-up work done over the following weeks needs to be coordinated to ensure that the development targets are achieved. This is especially true with respect to documentation. As one might expect, our observation from regularly checking in with the participants was that those activities that were closest to the participants’ research interests received the greatest follow-up attention.

It will come as no surprise that the participants identified much more work needing to be done than could be accomplished during a single five-day period. For this reason, we prefer to think of the hackathon as an ongoing project. The participating developers, who were chosen, in part, for their interest in having better glue code for phylogenetic analyses, will continue to work on the problems identified. In addition, other contributors to the respective software toolkits will be able to build upon the foundations and implementation examples that have been established. Future activities at NESCent (and hopefully elsewhere) will allow a continuation of the valuable face-to-face interactions. NESCent now offers an annual summer course on Computational Phyloinformatics to impart the programming skills necessary to take full advantage of the tools described here and to develop them yet further (see http://www.nescent.org/summer_course). Several of the instructors are participants in the hackathon. Two participants, J. Stajich and A. Vilella, have developed a tutorial on how to implement a phylogenetic workflow in BioPerl based on their work at the hackathon (presented at the 2007 ISMB conference); the material from that tutorial is freely available (see http://hackathon.nescent.org/Phylohackathon_1#Tutorials). NESCent has also sponsored a number of hackathon participants as mentors in the Google Summer of Code™ program, which funds students to work for a three-month period on open source software projects under the direction of expert software developers (see http://phylosoc.nescent.org).

Based on our experience, we conclude that the following conditions can be important factors to the success of a hackathon: (i) the community can articulate driving scientific questions that are difficult to answer primarily because of technical or interoperability barriers; (ii) a critical mass of individuals with the necessary technical capabilities and the willingness to collaborate exists; (iii) an organizing body can provide the technical and organizational framework both before, during and after the event.

Our experience with this event has convinced us that a hackathon is a very effective, not to mention enjoyable, way to make a substantive impact on the informatics infrastructure of a target community. It creates glue code that can be freely reused and extended. In so doing, it improves the ability of researchers and developers in the community to build complex analyses out of the wealth of existing tools. Undoubtedly, there are other domains in evolutionary bioinformatics where a diverse group of software developers could have a major impact on interoperability through a concerted collaborative coding effort. NESCent would be happy to entertain proposals for future hackathons through the Center’s informatics whitepaper mechanism (see http://www.nescent.org/informatics/whitepapers.php).

## Figures and Tables

**Table 1.** Representative list of targets and accomplishments.

Development target	Accomplishments		
(i) Sequence family evolution	BioPerl:	Support for TribeMCL, QuickTree, ClustalW, Phylip, and PAML	*
	BioPerl, Biopython:	Support for dN/dS-based tests for selection in HyPhy	
	Biojava:	Parser for Phylip alignment format	
	BioRuby:	Support for T-Coffee, MAFFT, and Phylip	
(ii) Reconciling trees	BioPerl:	Support for NJTree	*
	Biopython:	Wrapper for Softparsmap	
	BioRuby:	Model for phylogenetic trees and networks with graph algorithms	
	BioSQL:	Model for phylogenetic trees and networks with optimization methods and topological queries	
(iii) Phylogenetic footprinting	BioPerl:	Support for Footprinter, PhastCons, and using ClustalW over a sliding window	*
	BioRuby:	Calculate total tree length	
(iv) Phylogeny inference on non-molecular characters	BioPerl:	Interoperability between Bio::Phylo and BioPerl APIs	
	BioRuby:	NEXUS-compliant data model and parser for PAUP and TNT results	*
(v) Estimate divergence times	BioPerl:	Draft design of r8s wrapper	
(vi) NEXUS compliance issues	Biojava:	Work on interoperability between Biojava and JEBL	
	Biojava, BioRuby:	Level II-compliant NEXUS parser	
	All:	Evaluated major APIs; proposed compliance levels; gathered test files exposing common errors; fixed compliance issues in NCL and Bio::NEXUS reference implementations, worked on integrating those into GARLI and BioPerl, respectively	*

* Fully achieved target workflows.

**Table 2.** WWW addresses (URLs) of software projects and other resources mentioned in the report.

Resource name	WWW address
Bio::NEXUS	http://sourceforge.net/projects/bionexus
Bio::Phylo	http://search.cpan.org/dist/Bio-Phylo
Biojava	http://biojava.org
BioPerl	http://bioperl.org
Biopython	http://biopython.org
BioRuby	http://bioruby.org
BioSQL	http://biosql.org
Cyberinfrastructure for Phylogenetic Research (CIPRES)	http://www.phylo.org
EvoInformatics Working Group	http://evoinfo.nescent.org
GARLI (Genetic Algorithm for Rapid Likelihood Inference)	http://www.bio.utexas.edu/faculty/antisense/garli/Garli.html
HyPhy (Hypothesis testing using phylogenies)	http://www.hyphy.org
ISMB Tutorial Material	http://jason.open-bio.org/Bioperl_Tutorials/ISMB2007
JEBL (Java Evol, Biology Library)	http://sourceforge.net/projects/jebl
NCL (NEXUS Class Library)	http://hydrodictyon.eeb.uconn.edu/ncl
NEXUS compliance issues	http://hackathon.nescent.org/Supporting_Nexus
NESCent Informatics whitepaper program	http://informatics.nescent.org/whitepapers.php
Open Source Initiative (OSI)	http://www.opensource.org
PAUP*	http://paup.csit.fsu.edu
Phyloinformatics Hackathon	http://hackathon.nescent.org/Phylohackathon_1
Phyloinformatics Summer Course	http://www.nescent.org/summer_course
Phyloinformatics Summer of Code	http://phylosoc.nescent.org
PhyloXML	http://www.phyloxml.org
Substitution model exchange format	http://hackathon.nescent.org/CharacterModel_Object_Mode
